# The Effect of Thyroid Lobe Volume on the Common Carotid Artery Blood Flow in Thyroidectomy Position

**DOI:** 10.3390/jcm14051743

**Published:** 2025-03-05

**Authors:** Neslihan Hatınoğlu, Basar Erdivanli

**Affiliations:** 1 Department of Anesthesiology and Reanimation, Faculty of Medicine, Karadeniz Technical University, 61080 Trabzon, Turkey; neslihanhatinoglu@gmail.com; 2 Department of Anesthesiology and Reanimation, Faculty of Medicine, Recep Tayyip Erdogan University, 53100 Rize, Turkey

**Keywords:** common carotid artery, common carotid artery blood flow, Doppler ultrasonography, thyroid volume, cerebral oxygenation

## Abstract

**Background/Objectives**: This study investigates the effect of thyroid lobe size on common carotid artery hemodynamics during thyroidectomy. While prior research has reported reduced carotid blood flow during the procedure, the impact of thyroid size remains unclear. We hypothesized that larger thyroid lobes may influence carotid flow dynamics via external compression. **Methods**: Adult patients undergoing elective thyroidectomy were prospectively included. Doppler ultrasonography measured carotid artery diameters and flow characteristics at three time points: before anesthesia induction, after induction, and after surgical positioning. Regional cerebral oximetry was recorded. Each carotid artery was analyzed separately. **Results**: Data from 202 carotid arteries (132 patients) were analyzed. Baseline carotid diameters and flow velocities were similar between patients with normal and large thyroid lobes. Anesthesia induction reduced flow velocities in all patients. After surgical positioning, patients with large thyroid lobes had significantly increased peak systolic velocity, leading to an overestimation of carotid blood flow, when using formula-based calculations. Manually traced Velocity Time Integral confirmed the increase in peak systolic velocity and a shortened systolic/diastolic ratio in these patients. Receiver operating characteristic analysis identified a thyroid lobe volume cutoff of 19.7 mL (AUC: 0.93, Sensitivity: 85%, Specificity: 98%). Regional cerebral oxygen saturation remained unchanged (*p* > 0.05). **Conclusions**: Larger thyroid lobes are associated with altered carotid flow dynamics during thyroidectomy, emphasizing diastolic flow. While these findings provide insight into thyroid-related hemodynamic changes, their applicability to patients with pre-existing carotid stenosis or peripheral artery disease remains uncertain, as our study population did not include such cases.

## 1. Introduction

Cardiovascular diseases remain a global public health concern, particularly disorders of cerebral circulation. Recent studies have documented that even subtle changes in thyroid function can carry implications for vascular outcomes. For instance, Groothof et al. reported that small, within-reference-range alterations in thyroid hormone levels were linked to higher cardiovascular mortality [1], while Paschou et al. reviewed how hyper- and hypothyroidism—both clinical and subclinical—can modulate vascular stiffness and cardiac function [2]. Beyond thyroid hormone status, morphological characteristics of the thyroid gland have also been implicated in subclinical vascular changes. Two separate works by Jakubiak et al. investigated euthyroid subjects, showing that thyroid volume may correlate with measures such as intima-media thickness and ankle-brachial index [3,4]. Taken together, these findings indicate that the thyroid gland’s structural and functional parameters may influence cardiovascular hemodynamics, even without overt thyroid dysfunction.

Despite this growing evidence, few studies have examined how these thyroid-related vascular influences might manifest in the perioperative setting. Thyroidectomy requires a semi-Fowler position with the head extended—a posture that can alter cervical geometry and potentially affect regional blood flow. Some studies have reported reduced carotid blood flow and cerebral oxygenation beginning with the positioning, continuing until the end of surgery [5,6], but none have specifically addressed the impact of thyroid lobe size, leaving this relationship unexplored. Patients undergoing thyroidectomy frequently present with enlarged lobes due to diverse indications such as pressure symptoms, voice changes, cosmetic concerns, hyperthyroidism, or malignancy, some intricately tied to larger thyroid lobes. However, it is not clear whether these anatomical variations amplify any positioning-related hemodynamic changes. This diversity remains largely unexplored in prior research. Given the potential of a large thyroid lobe to compress the common carotid artery [7], we recognize a gap in our understanding. Considering the prevalence of thyroid nodules detected during carotid Color Doppler Ultrasound [8], it is plausible that an enlarged thyroid lobe—by virtue of its location—could affect blood flow through external compression. However, to date, such a potential mechanism has remained largely unexplored in the surgical context.

We hypothesized that a larger-than-normal thyroid lobe, influenced by its anatomical relationship, may exert compression on the common carotid artery, potentially impacting blood flow dynamics. To test this hypothesis, we planned a prospective, observational study, examining changes in carotid blood flow under surgical positioning. This study aimed to investigate the alterations in the common carotid artery induced by head positioning during thyroidectomy. We particularly focused on the role of thyroid lobe size in modifying carotid blood flow dynamics, assessing whether an enlarged thyroid gland could intensify any positioning-related hemodynamic changes. By clarifying this interaction, we hoped to determine whether a large thyroid lobe might pose distinct perioperative risks for carotid flow while recognizing that broader thyroid morphology/function considerations may also play a role.

## 2. Materials and Methods

Adult patients scheduled for elective thyroidectomy were prospectively enrolled if they were ≥18 years of age and provided written informed consent. Patients who were pregnant, in emergency conditions, or had a history of subtotal thyroidectomy, documented allergies to anesthetic agents, anemia, labile hypertension, or abnormal thyroid hormone levels indicating undertreatment of thyroid pathology were excluded. If ultrasound examination was not feasible on one side, only that side’s data were excluded from the analysis, while contralateral measurements were retained.

This prospective, observational study was registered in clinicaltrials.gov (trial registry number NCT06797388) and conducted at a tertiary hospital between 6 March 2017 and 20 March 2020.

The primary outcomes of this study were to evaluate the relationship between thyroid lobe volume and alterations in the systolic/diastolic flow time ratio during head extension for thyroidectomy and to identify a thyroid lobe volume cutoff associated with a significant change through post hoc analysis. Secondary outcomes included assessing changes in carotid artery blood flow and regional cerebral oxygenation at three key time points: baseline (T0), post-anesthesia induction (T1), and post-surgical positioning (T2). These outcomes aim to elucidate the hemodynamic effects of thyroid lobe volume on carotid artery dynamics and brain perfusion during surgery.

### 2.1. Monitorization and Baseline Measurements

**Figure 1:**
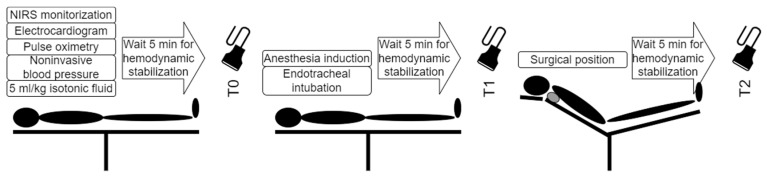
Timeline of data collection.

Time points and measurements are shown in Figure 1. Briefly, patients were taken into the surgical room without premedication. In addition to the routine monitorization (electrocardiogram, noninvasive blood pressure, pulse oximetry), regional cerebral oximetry (INVOS 5100C, Medtronic, Minneapolis, MN, USA) was applied to the forehead [9]. Baseline hemodynamic data were recorded. Diameters and blood flow characteristics were measured with Doppler ultrasound (Esaote MyLab Five, Genoa, Italy; LA435 Linear probe) and recorded (T0).

### 2.2. Anesthesia İnduction

Following 4 mL/kg intravenous (iv) hydration with isotonic saline infusion, anesthesia was induced with 0.02 mg/kg iv midozolam, 2 mcg/kg of iv fentanyl, and 2 mg/kg of iv propofol. Muscle relaxation was obtained with 0.6 mg/kg iv rocuronium. An infusion of 0.1 mcg/kg/min remifentanil was started to maintain hemodynamic stability. Due to the known effect of hyperoxia on cerebral blood flow, preoxygenation was performed with 40% FiO_2_ [10]. In addition, tidal volume and respiratory rate were maintained within age-appropriate values. Anesthesia was maintained with a maximum inspiratory concentration of 2% sevoflurane and 0.1–0.3 mcg/kg/min remifentanil infusion. Oxygenation was maintained with 2 L/min fresh gas flow and 40% FiO_2_. Doppler ultrasound measurements were performed after the tracheal intubation (T1) and after the surgical positioning (T2).

### 2.3. Color Doppler Ultrasound Measurements

All measurements were performed by the same researcher using an LA435 short linear probe and carotid mode. Briefly, a B-mode image of the carotid artery was obtained at the level of thyroid cartilage [11]. The probe was then slid caudally to inspect the common carotid artery for gross lesions and to visualize the caudal portion of the carotid bulb. Diameters of the common carotid artery at the end of systole (CCA-SD) and end of diastole (CCA-DD) were measured 2 cm caudal to the bulb. Color Flow Mapping and Pulsed Wave options were activated to measure peak systolic velocity (PSV) and end-diastolic velocity (EDV). These values were used to calculate the mean velocity (MV):

(1) $$MV=\frac{PSV+2\times EDV}{3}$$

(2) $$Gosling\;pulsatility\;index\;(PI)=\frac{PSV-EDV}{MV}$$

(3) $$Pourcelot\;resistance\;index\left(RI\right)=\frac{PSV-EDV}{PSV}$$

Calibration of the Doppler ultrasound device was performed according to the manufacturer’s guidelines to ensure measurement accuracy.

### 2.4. Calculation of Common Carotid Artery Blood Flow

Initially, the Velocity Time Integral (VTI) was calculated as:

(4) VTI=MV×Gosling PI

Carotid artery blood flow was then calculated with the following formula [12]:

(5) $$\mathrm{Carotid}\;\mathrm{artery}\;\mathrm{blood}\;\mathrm{flow}=\pi \times \frac{\left(carotid\;inner\;diameter\right)2}{4}\times VTI\times Heart\;rate$$

During data analysis, cases with altered systolic/diastolic flow ratios were identified. To ensure the precision of the VTI measurements in these cases, manual VTI tracing was subsequently performed. This approach allowed us to address the potential inaccuracies from automated calculations and ensure that the blood flow was measured with optimal precision in these patients.

### 2.5. Methods Against Bias

This was an observational study with no randomization. A radiologist performed thyroid-lobe ultrasound measurements and did not share these findings with the primary investigator, who conducted the carotid Doppler measurements, thus avoiding the assignment of “large” vs. “normal” lobes during enrollment or data collection. An independent radiology technician then selected Doppler waveforms and calculated the carotid diameters and velocities. The data analyst was blinded to whether patients had normal or large thyroid lobes, and the investigators did not take part in anesthesia management. The dataset was anonymized, and the final classification threshold for thyroid lobe size was determined only after all measurements had been completed.

### 2.6. Statistical Analysis

Statistical analysis was performed with R (version 4.2.2; R Foundation for Statistical Computing, Vienna, Austria). A priori sample size was calculated as 204 with an effect size of 0.35, a type I error of 0.05, a power of 0.8, and a one-tailed α of 0.05 (G*Power version 3.1.9.7). Given that both carotid arteries were analyzed separately, a minimum of 108 patients were required to account for an anticipated 5% rate of incomplete records.

To ensure adequate representation of patients with larger thyroid lobes, a minimum of 39 patients was targeted in this group. This threshold was selected based on achieving at least 80% power to detect a clinically meaningful difference in peak systolic velocity (derived from preliminary data). The final cohort comprised 101 patients with normal thyroid lobes and 39 patients with larger thyroid lobes, yielding an allocation ratio of approximately 2.6:1.

Patients were divided into two groups according to gender-specific thresholds for normal thyroid volume (15 mL for adult females, 18 mL for adult males) [13]. Prior to selecting statistical tests, the distribution of each continuous variable was examined using the Shapiro–Wilk test. Because most variables did not follow a normal distribution, continuous variables were expressed as medians (interquartile ranges), and between-group comparisons were performed using the Kruskal–Wallis test followed by Dunn’s post hoc analysis. Levene’s test was used to assess the homogeneity of variance between groups. Outliers were identified using the modified Z-Score method (|M_z| > 3.5). Categorical variables were analyzed using the Chi-square test.

To ensure precision in cases with altered systolic/diastolic flow ratios, carotid artery blood flow was recalculated using manually traced VTI data. Demographic properties, vital signs, regional cerebral oximetry values, and ultrasonographic findings were summarized with descriptive statistics. No interobserver variability analysis was necessary, as all measurements were performed by the same researcher.

Receiver operating analysis (ROC) was used to determine the cutoff point of thyroid lobe volume to identify a ratio of systolic/diastolic flow time < 0.3 (cutpointr package, version 1.1.2, maximize_metric function). A *p*-value of <0.05 was considered statistically significant throughout.

## 3. Results

**Figure 2:**
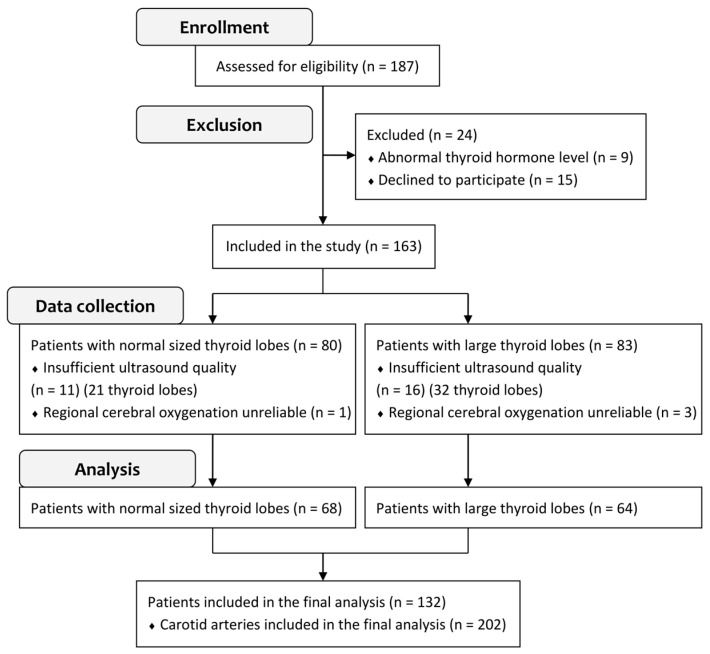
Consort diagram illustrating participant recruitment, exclusions, and study completion.

Data from a total of 202 measurements obtained from 132 patients were analyzed (Figure 2). The patient characteristics are summarized in Table 1. The single patient characteristic significantly associated with a large thyroid lobe size was male gender (*p* = 0.02).

Values measured by Doppler ultrasound and derived by formulas are presented in Table 2.

### Threshold Analysis of Systolic/Diastolic Flow Time Ratios

**Figure 3:**
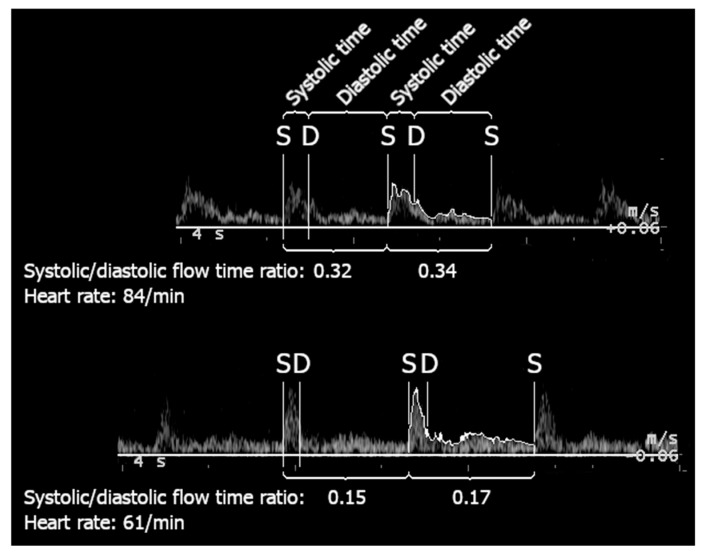
Example of velocity time integral (VTI) trace analysis with systolic and diastolic time annotations. The top panel illustrates a trace with a systolic/diastolic flow time ratio of 0.32 at a heart rate of 84 beats per minute, while the bottom panel shows a trace with a lower ratio of 0.15 at a heart rate of 61 beats per minute. Systolic (S) and diastolic (D) phases are labeled for clarity, and the corresponding time intervals are highlighted. These traces demonstrate how systolic and diastolic flow times are measured to calculate the systolic/diastolic flow time ratio used in the study.

To ensure accuracy, Doppler ultrasound images were systematically assessed for flow characteristics. In cases of large thyroid lobes, systolic flow time was significantly reduced, favoring diastolic flow time (Figure 3). The median systolic/diastolic flow time ratio was significantly lower in patients with large thyroid lobes compared to those with normal lobes (0.19 vs. 0.35, *p* = 0.01, 95% CI: 0.019–0.154). Subsequently, carotid artery blood flow was recalculated using manually traced VTI data. Manual VTI tracing was performed to avoid potential inaccuracies from automated VTI calculations, particularly in cases with altered systolic/diastolic flow ratios, ensuring that blood flow was measured with optimal precision. Based on these manually traced values, no significant difference in carotid artery blood flow was observed between the groups.

**Figure 4:**
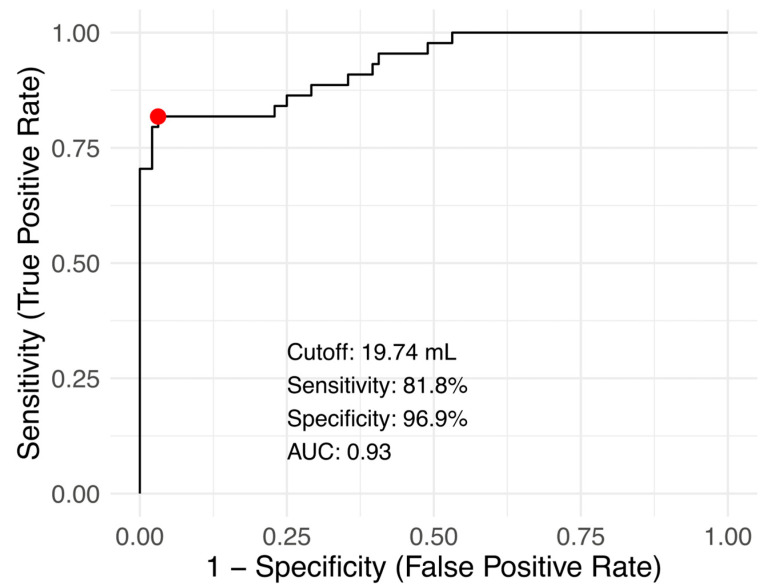
Receiver operating characteristic (ROC) curve for determining the optimal thyroid lobe volume cutoff to identify a systolic/diastolic flow time ratio <0.3. The ROC analysis yielded an optimal thyroid lobe volume cutoff of 19.7 mL, with an area under the curve (AUC) of 0.93. At this threshold, the sensitivity was 81.82%, specificity was 96.88%, and overall accuracy was 92.14%. The curve demonstrates the trade-off between sensitivity and specificity, with the optimal point marked on the curve.

Receiver operating analysis to determine the optimal thyroid lobe volume to identify a systolic/diastolic flow time ratio <0.3 yielded the optimal cutoff point as 19.7 mL with area under the curve: 0.93, sensitivity: 81.8%, specificity: 96.9%, accuracy: 92.1% (Figure 4).

**Figure 5:**
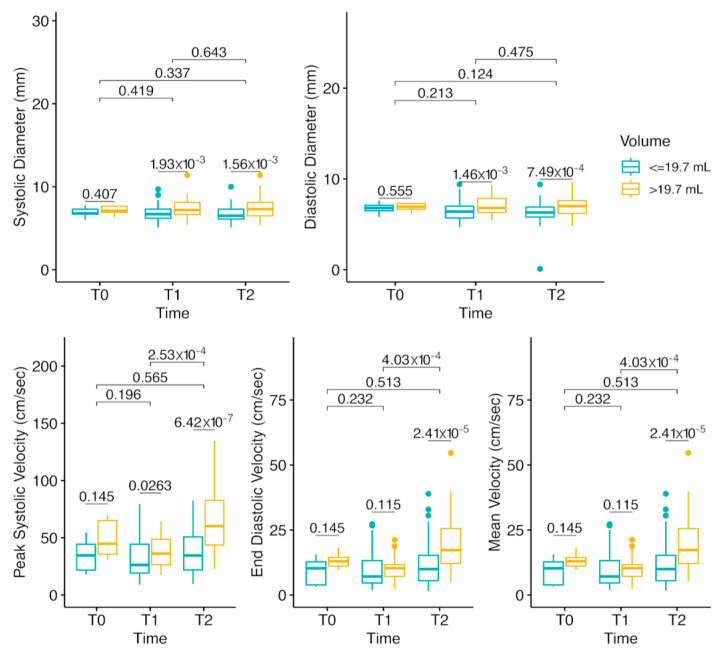
Boxplots show common carotid artery systolic diameter, diastolic diameter, peak systolic velocity (PSV), end-diastolic velocity (EDV), and mean velocity (MV) across different time points (T0, T1, T2) in patients with thyroid lobes ≤19.7 mL and >19.7 mL. Statistical comparisons within groups (*p*-values) are displayed, with Kruskal–Wallis tests used for overall group comparisons. Error bars indicate interquartile ranges. Large interquartile ranges in PSV, EDV, and MV at T2 primarily reflect inter-patient variability, with additional influence from a single extreme outlier per parameter.

**Figure 6:**
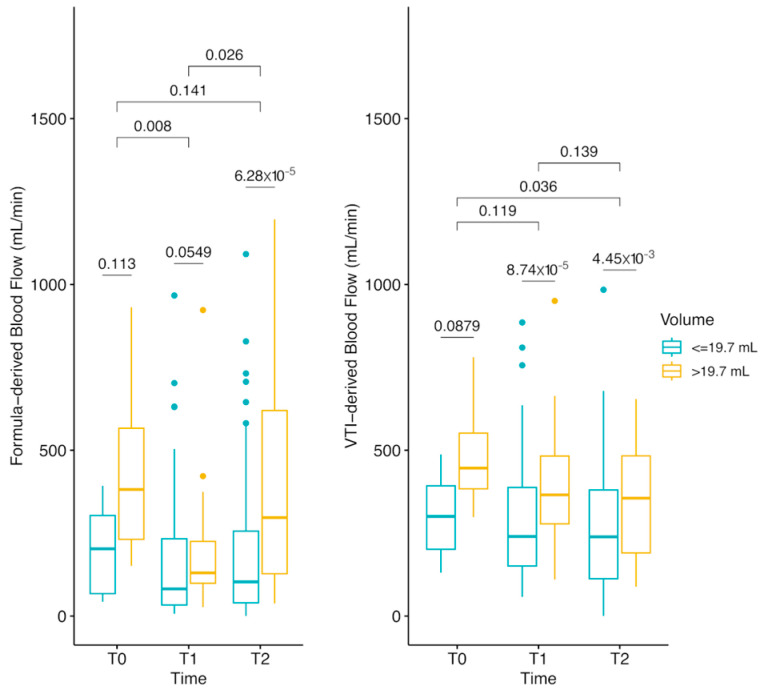
Formula-derived and VTI-derived carotid blood flow at three time points during thyroidectomy (T0: baseline, T1: post-anesthesia induction, T2: post-surgical positioning), stratified by thyroid lobe volume (>19.7 mL vs. ≤19.7 mL). The left panel shows carotid blood flow (mL/min) calculated using PSV, EDV, and heart rate, which increased significantly after surgical positioning in patients with large thyroid lobes. The right panel illustrates carotid blood flow measured using VTI tracing. Formula-based calculations suggested an increase in blood flow due to elevated peak systolic velocity (PSV) in the large thyroid group, but VTI-derived measurements confirmed no significant difference in total blood flow, as systolic duration was significantly shorter in these patients. Statistical comparisons are indicated for each time point, with overall Kruskal–Wallis test results provided at the top. Error bars represent interquartile ranges, and group stratification illustrates differences based on thyroid lobe volume.

Doppler measurements stratified according to the calculated cutoff thyroid lobe volume are shown in Figure 5 and Figure 6. Briefly, baseline carotid artery diameters and flow velocities were similar between the two thyroid lobe size groups. After anesthesia induction, no significant change in common carotid artery diameters was observed, though a modest but non-significant increase in flow velocities occurred. In the large thyroid group, surgical positioning caused significant increases in peak systolic velocity (PSV), end-diastolic velocity (EDV), and mean velocity (MV), along with a slight but significant reduction in common carotid artery diameters during both systole and diastole. Inter-patient variability was the primary contributor to the wide spread observed in PSV, EDV, and MV at T2, with a single extreme outlier detected per parameter.

After surgical positioning, the formula-derived carotid blood flow (Figure 6, left panel) was significantly higher in patients with large thyroid lobes (>19.7 mL) due to elevated PSV. However, VTI-derived blood flow (Figure 6, right panel) remained stable, as systolic duration was significantly shorter in the large thyroid group.

## 4. Discussion

This study demonstrates that an enlarged thyroid gland may alter common carotid artery characteristics, specifically by decreasing systolic flow time, without significantly affecting overall carotid artery blood flow or regional cerebral oxygen saturation obtained from the frontal lobe. Similar to the subclinical correlations reported by Jakubiak et al. [3,4], which emphasized associations between thyroid volume and peripheral vascular indices in euthyroid populations, our perioperative data suggest that gland size alone can influence vascular dynamics—albeit through a potentially distinct, mechanical compression effect. The combination of head extension and an enlarged thyroid gland was found to reduce systolic flow time, with a compensatory emphasis on diastolic flow.

The thyroid lobe and common carotid artery are anatomically related to such an extent that carotid pulsations allow examination of thyroid lobe elasticity [14,15]. Previous studies have documented the impact of neck hyperextension in eliciting carotid sinus hypersensitivity [16,17]. Additionally, case reports have described direct external compression of the carotid artery by thyroid cysts, leading to transient neurological deficits, further supporting the role of thyroid–vascular interactions in flow regulation [18,19]. The shift in flow dynamics, as observed in this study, may result from increased external pressure exerted by the large thyroid lobe on the common carotid artery, consistent with the Windkessel effect. As systolic blood flow encounters resistance due to this compression, the diastolic phase allows for the propulsion of the blood toward the head via arterial elastance. While prior literature mostly addresses cystic or nodular masses [7,20], our findings highlight that even a generally enlarged gland can produce comparable flow alterations when combined with head extension.

Recent evidence suggests that morphological and functional thyroid changes can influence vascular parameters outside the operative setting. In euthyroid populations, enlarged thyroid volume has been associated with higher intima-media thickness and altered peripheral indices [3,4], while subclinical variations in thyroid hormones have correlated with increased cardiovascular risk [1,2]. Although these studies have not examined direct mechanical compression during thyroidectomy, they highlight a broader concept that both the size of the gland and subtle thyroid function shifts might affect vascular dynamics. Our findings add an intraoperative perspective to this literature, indicating that an enlarged thyroid lobe, under head extension, can shorten systolic flow time but preserve overall flow via compensatory diastolic enhancement.

Another important regulatory mechanism is the baroreflex response to changes in carotid artery flow. Previous studies have shown that external compression of the carotid artery can trigger baroreceptor activation and alter autonomic control of blood flow, as demonstrated in a study using a neck compression collar, which led to increased systolic blood pressure and prolonged pressure recovery time during Valsalva maneuvers [21]. Additionally, the carotid sinus nerve plays a critical role in modulating baroreflex sensitivity and maintaining hemodynamic stability, adjusting autonomic tone to compensate for external vascular compression [22]. This aligns with our findings, where increased PSV in patients with large thyroid lobes may stimulate carotid baroreceptors, leading to vagally mediated bradycardia. This, in turn, shortens systolic flow time while prolonging the diastolic phase. This physiological compensation explains why VTI-derived total carotid blood flow remained stable despite the increased PSV. As our measurements were limited to the common carotid artery near the bulb, the area directly adjacent to the thyroid lobe remained unexplored. Consequently, the proposed mechanism remains speculative without further direct evidence. However, studies in baroreflex activation therapy for resistant hypertension have shown that manipulating the carotid region can meaningfully lower blood pressure and heart rate by engaging baroreceptors [23], underscoring how mechanical stimuli to this area can have systemic hemodynamic effects.

This study did not identify any additional patient characteristics linked to the aforementioned alterations in blood flow. While the reference limits for a normal thyroid lobe were taken from an American study [13], other publications suggest that thyroid lobe volume correlates with age and body mass index [24]. However, in our dataset, thyroid lobe volume was only associated with gender. Our perioperative setting may partly explain the absence of further demographic associations, contrasting studies like Groothof et al. [1] that found age- or BMI-linked variations in thyroid function impacting longer-term vascular outcomes. Here, the mechanical effect under anesthesia and positioning may overshadow such baseline factors.

Despite the majority of patients being over 45 years old, a demographic associated with an increased risk of thyroid cancer, we did not identify any instances of carotid artery stenosis or plaque. Baseline common carotid artery blood flow values fell within normal limits [25]. The absence of carotid artery plaques in our study population likely explains the stability of regional cerebral oxygen saturation throughout the procedure. In patients with carotid plaques or stenosis, these values could have been influenced by impaired cerebral perfusion. Likewise, the subclinical relationships documented in earlier investigations [1] might manifest differently in a population with established vascular disease. Therefore, our findings may not be generalizable to high-risk populations with vascular disease, such as patients with peripheral artery disease or cardiac conditions. Future studies should explore how vascular disease may alter carotid flow dynamics and cerebral oxygenation in these populations.

When the stenosis is located caudal to the thyroid lobe, the cranial portion of the artery may receive inadequate blood flow, rendering arterial elastance ineffective for blood storage during systole. In cases where the contralateral carotid artery and vertebral arteries remain healthy, they may compensate. However, bilateral carotid stenosis would significantly affect blood flow and warrant more cautious management.

In situations where external pressure is applied to the carotid artery, the significance of diastolic flow becomes particularly critical, as in the case of an enlarged thyroid lobe. The baroreflex response may lead to compensatory bradycardia, reducing systolic duration while preserving cerebral perfusion through prolonged diastolic flow. A recent meta-analysis of carotid sinus massage as a vagal maneuver [26] reinforced that direct carotid pressure can terminate arrhythmias by activating the baroreceptor reflex and prolonging diastole, conceptually paralleling our observation that an enlarged thyroid lobe can shift systolic/diastolic flow balance. Finally, reduced baroreflex sensitivity and increased arterial stiffness have been implicated in exaggerated blood pressure responses under mechanical or physiological stress [27]. Although our population lacked clinically evident atherosclerosis, this concept parallels our observation that local external pressure—in this case, thyroid lobe enlargement—can modify normal flow responses. In such patients, careful consideration is needed when administering cardioactive drugs, as bradycardia and tachycardia have opposing effects on diastolic filling time. Bradycardia may be beneficial in maintaining cerebral perfusion, whereas tachycardia could shorten diastolic time and potentially impair cerebral perfusion.

This study has several limitations. The upward position of the carotid bulb is commonly encountered and presents a routine challenge in ultrasound examination of the carotid artery, necessitating techniques to interpret internal carotid artery blood flow from the main carotid artery blood flow traces [28]. Therefore, this pilot study focused solely on the common carotid artery blood flow, and no measurements were taken at the internal or external carotid arteries. Additionally, vertebral arteries, which are a significant contributor to cerebral blood flow, were not visualized. Although the literature suggests that vertebral arteries are mainly affected by head rotation [29], future studies should assess their role in cerebral blood flow dynamics during thyroid surgery. Moreover, the choice to exclude patients with abnormal thyroid hormone levels may limit the generalizability of these findings. To ensure accurate blood flow measurements in patients with altered systolic/diastolic flow ratios, manual VTI tracing was performed, further reinforcing the reliability of our data.

Our study also has notable strengths. We employed a prospective design with real-time ultrasound assessments and included a range of thyroid lobe sizes, from normal to markedly enlarged, which enabled a focused investigation of mechanical effects. Additionally, all Doppler measurements were performed by a single operator, minimizing interobserver variability, and manual VTI tracing provided a more precise assessment of complex flow patterns. These methodological choices enhance the robustness of our findings and underscore the potential for enlarged thyroid lobes to influence perioperative carotid hemodynamics.

A comprehensive follow-up study is warranted to assess internal and external carotid artery blood flow, vertebral artery stenosis, and factors such as thyroid lobe size, thyroid hormones, and the impact of the superior thyroid artery on thyroid perfusion.

Finally, the characteristics of the thyroid lobe (stiffness, calcification, contour) and the anatomy of the neck (circumference, muscularity) are potential determinants of the degree of pressure applied to the common carotid artery. Evaluating these factors would require a detailed and time-consuming procedure. While this study adopted a pragmatic approach, we believe that this limitation does not compromise the validity of our findings.

## 5. Conclusions

This study demonstrates that an enlarged thyroid lobe may significantly affect common carotid artery flow dynamics by reducing systolic flow time and emphasizing diastolic flow, yet overall carotid blood flow and regional cerebral oxygen saturation remain unchanged. Although future investigations are needed to determine whether these positional effects carry clinical implications in patients with vascular disease or more advanced thyroid pathology, our findings underscore the potential role of thyroid lobe size in perioperative carotid hemodynamics.

## Figures and Tables

**Table 1:** Baseline characteristics of patients stratified by thyroid lobe size (normal vs. large). Patient characteristics are presented as mean ± standard deviation, number (percentage), or median (interquartile range).

	Thyroid Lobe Size		
Characteristic	Normal (n = 68)	Large (n = 64)	*p*-Value
Age, years	46.6 ± 11.1	52 ± 10.3	0.015
Sex (female), n (%)	42 (62%)	35 (55%)	0.7
BMI, kg/m^2^	28.94 ± 3.61	29.12 ± 3.58	>0.9
ASA Physical Score			0.7
1	34 (50%)	36 (56%)	
2	34 (50%)	28 (44%)	
Hemoglobin, g/dL	12.90 ± 1.12	13.76 ± 1.53	>0.9
TSH, mIU/	1.87 ± 1.04	1.37 ± 1.01	>0.9
Free T3, ng/dL	2.77 ± 0.73	3.00 ± 0.25	>0.9
Free T4, µg/dL	1.02 ± 0.15	1.05 ± 0.21	>0.9
Thyroid lobe volume, mL	7.54 (4.8–10.1)	22.8 (18.4–32.4)	<0.001

BMI, body mass index; ASA, American Society of Anesthesiologists.

**Table 2:** Hemodynamic indices. Data are presented as mean ± standard deviation or median (interquartile ranges).

	Time Points			*p*-Value	
	T0 (n = 132)	T1 (n = 132)	T2 (n = 132)	*p*-Value (All Time Points)	*p*-Value (T1 vs. T2)
SBP, mmHg	127.6 ± 11.2	96.5 ± 9.8	99.9 ± 9.5	<0.001 *	0.865
MBP, mmHg	87.8 ± 12.2	70.9 ± 4.5	78.8 ± 2.5	<0.001 *	0.319
Heart rate, beats/min	74.3 ± 11.6	73.1 ± 8.3	67.9 ± 7.2	0.552 *	0.528
FiO_2_, %	Room air	40 ± 0.8	39 ± 1.3	NA	>0.9
SrcO2, %	70.2 ± 10.6	70.1 ± 10.6	70.4 ± 8.5	>0.9 *	>0.9
CCA-SD, mm	7.02 ± 0.56	6.93 ± 1.02	6.89 ± 1.06	>0.9 *	0.678
CCA-DD, mm	6.78 ± 0.54	6.62 ± 0.93	6.52 ± 1.13	0.654 *	0.432
PSV, cm/s	36.6 (26.3–50.5)	31.7 (20.7–44.5)	40.7 (24.1–63.3)	0.004 **	0.001
EDV, cm/s	11.1 (9–13.5)	8 (5–12.3)	12 (6.3–18.2)	0.003 **	0.001
MV, cm/s	21.6 (14.8–25.4)	15.9 (10.2–23.5)	21.7 (12.8–33)	0.004 **	<0.001
Gosling PI	36.3 (26–50)	23.9 (17.1–39.7)	32 (16.6–45)	0.082 **	0.205
Pourcelot RI	26.25 (26–50)	24 (17–39.7)	26.3 (16.5–44.9)	0.082 **	0.207
Blood flow, mL/min					
Formula-derived	478 (247–726)	205 (79–464)	307.8 (95–750)	<0.001 **	<0.001
VTI-derived	558 (430–717)	427 (260–633)	377 (190–639)	0.343 **	0.631

T0: Baseline; T1: After tracheal intubation; T2: After surgical positioning; SBP: Systolic arterial blood pressure; MBP: Mean arterial blood pressure; FiO_2_: Fraction of inspired oxygen concentration, SrcO2: Regional cerebral oxygen saturation; CCA-SD: Common carotid artery systolic diameter; CCA-DD: Common carotid artery diastolic diameter; PSV: Peak systolic velocity; EDV: End-diastolic velocity; MV: Mean velocity; PI: Pulsatility index; RI: Resistance index; VTI: Velocity Time Integral; NA: Not applicable; * Analysis of Variance; ** Kruskal–Wallis test.

## Data Availability

The raw data supporting the reported results and the R source files used during the analysis will be made available by the authors on reasonable request.

## Abbreviations

The following abbreviations are used in this manuscript:

CCA-SD	Diameter of the common carotid artery at the end of systole
CCA-DD	Diameter of the common carotid artery at the end of diastole
PSV	Peak systolic velocity
EDV	End-diastolic velocity
MV	Mean velocity
PI	Gosling pulsatility index
RI	Pourcelot resistance index
VTI	Velocity time integral
BMI	Body mass index
